# Radiation dosimetry of the α_4_β_2_ nicotinic receptor ligand (+)-[^18^F]flubatine, comparing preclinical PET/MRI and PET/CT to first-in-human PET/CT results

**DOI:** 10.1186/s40658-016-0160-5

**Published:** 2016-10-21

**Authors:** Mathias Kranz, Bernhard Sattler, Solveig Tiepolt, Stephan Wilke, Winnie Deuther-Conrad, Cornelius K. Donat, Steffen Fischer, Marianne Patt, Andreas Schildan, Jörg Patt, René Smits, Alexander Hoepping, Jörg Steinbach, Osama Sabri, Peter Brust

**Affiliations:** 1 Institute of Radiopharmaceutical Cancer Research, Research Site Leipzig, Helmholtz-Zentrum Dresden-Rossendorf, Permoserstraße 15, 04318 Leipzig, Germany; 2 Department of Nuclear Medicine, University Hospital Leipzig, Leipzig, Germany; 3 ABX advanced biochemical compounds Ltd., Radeberg, Germany; 4 Division of Brain Sciences, Department of Medicine, Hammersmith Hospital Campus, Imperial College London, London, UK

**Keywords:** Image-based internal dosimetry, (+)-[^18^F]flubatine, Preclinical hybrid PET/MRI, Radiation safety, Nicotinic receptors, Dosimetry, OLINDA/EXM

## Abstract

**Background:**

Both enantiomers of [^18^F]flubatine are new radioligands for neuroimaging of α_4_β_2_ nicotinic acetylcholine receptors with positron emission tomography (PET) exhibiting promising pharmacokinetics which makes them attractive for different clinical questions. In a previous preclinical study, the main advantage of (+)-[^18^F]flubatine compared to (−)-[^18^F]flubatine was its higher binding affinity suggesting that (+)-[^18^F]flubatine might be able to detect also slight reductions of α_4_β_2_ nAChRs and could be more sensitive than (−)-[^18^F]flubatine in early stages of Alzheimer’s disease. To support the clinical translation, we investigated a fully image-based internal dosimetry approach for (+)-[^18^F]flubatine, comparing mouse data collected on a preclinical PET/MRI system to piglet and first-in-human data acquired on a clinical PET/CT system. Time-activity curves (TACs) were obtained from the three species, the animal data extrapolated to human scale, exponentially fitted and the organ doses (OD), and effective dose (ED) calculated with OLINDA.

**Results:**

The excreting organs (urinary bladder, kidneys, and liver) receive the highest organ doses in all species. Hence, a renal/hepatobiliary excretion pathway can be assumed. In addition, the ED conversion factors of 12.1 μSv/MBq (mice), 14.3 μSv/MBq (piglets), and 23.0 μSv/MBq (humans) were calculated which are well within the order of magnitude as known from other ^18^F-labeled radiotracers.

**Conclusions:**

Although both enantiomers of [^18^F]flubatine exhibit different binding kinetics in the brain due to the respective affinities, the effective dose revealed no enantiomer-specific differences among the investigated species. The preclinical dosimetry and biodistribution of (+)-[^18^F]flubatine was shown and the feasibility of a dose assessment based on image data acquired on a small animal PET/MR and a clinical PET/CT was demonstrated. Additionally, the first-in-human study confirmed the tolerability of the radiation risk of (+)-[^18^F]flubatine imaging which is well within the range as caused by other ^18^F-labeled tracers. However, as shown in previous studies, the ED in humans is underestimated by up to 50 % using preclinical imaging for internal dosimetry. This fact needs to be considered when applying for first-in-human studies based on preclinical biokinetic data scaled to human anatomy.

**Electronic supplementary material:**

The online version of this article (doi:10.1186/s40658-016-0160-5) contains supplementary material, which is available to authorized users.

## Background

For neuroimaging of α_4_β_2_ nicotinic acetylcholine receptors (nAChRs), a new promising radioligand [^18^F]flubatine, formerly called [^18^F]NCFHEB, was recently discovered [1]. The α_4_β_2_ receptor subtype comprises ~90 % of all nAChRs in the brain [2] and is involved in different neuronal functions and diseases like Parkinson’s disease [3], schizophrenia [4], and Alzheimer’s disease (AD) [5]. Its density is reduced in post mortem studies of patients who suffered from AD throughout the cerebral cortex [6, 7]. The possibility of detecting disease-related alterations of nAChRs might provide important information on the transformation from mild cognitive impairment to AD [8, 9]. Most of other tracers targeting nAChRs, e.g., 2-[^18^F]fluoro-A-85380 [10] or [^18^F]nifene [11] have the drawback of long acquisition times or a low specific binding. The enantiomers (−)-[^18^F]flubatine and (+)-[^18^F]flubatine have a high affinity (Ki = 0.112 nM, Ki = 0.064 nM, respectively) and selectivity [12] to α_4_β_2_ receptors as demonstrated in mice [12], piglets [13], and rhesus monkeys [14]. Although both enantiomers are suitable for clinical application, the (−) enantiomer was chosen first due to faster binding kinetics in pig brains [15] for an early clinical study [16]. As expected, (−)-[^18^F]flubatine showed fast kinetics in humans reaching its pseudo equilibrium within 90 min p.i. in the thalamus and within 30 min p.i. in all cortical regions [7, 15]. Metabolite analysis have shown that (−)-[^18^F]flubatine has a high stability after i.v. injection in patients with AD and healthy controls. More than 85 % of unchanged tracer was found at 90 min p.i. determined by dynamic blood sampling followed by radio-HPLC [17]. However, a metabolite correction of the positron emission tomography (PET) data is inevitable [17]. In the previous preclinical study, the main advantage of (+)-[^18^F]flubatine compared to (−)-[^18^F]flubatine was its higher binding affinity [13]. Therefore, the data suggest that (+)-[^18^F]flubatine might be able to detect also slight reductions of α_4_β_2_ nAChRs and could be more sensitive than (−)-[^18^F]flubatine in early stages of AD.

For the translation of newly developed radiotracers into clinical study phases, a radiation dosimetry, i.e., the calculation of the absorbed organ and effective dose in humans is mandatory. These dose assessments are mainly based on biokinetic data obtained in small animals prior to permission for first-in-human use [18]. The human safety and tolerability has to be shown in a whole-body biodistribution and dosimetry study. To determine the biodistribution of a tracer, terminal methods (sacrificing the animals followed by organ harvesting) or non-invasive methods using quantitative molecular imaging techniques like PET and single-photon emission computed tomography (SPECT) can be applied [19]. Imaging-based methods should be preferred in order to minimize the necessary number of laboratory animals and the duration of the investigation. Constantinescu et al. [20, 21] has shown the feasibility of a preclinical assessment of the radiation dose in humans by radiotracers using mice and rats on a small animal PET/CT. However, since the soft tissue contrast is comparatively poor in CT, the organ delineation remains difficult.

In this work, we introduce our preclinical PET/MRI system to estimate the absorbed radiation dose in humans based on small animal quantitative PET data and evaluate the feasibility and suitability of the proposed protocol. Additionally, piglets are used to investigate the influence of the body size on the dosimetry result. Subsequently, we report on the first-in-human internal dosimetry using (+)-[^18^F]flubatine obtained in three healthy volunteers. We compare the results of the image-based approach after i.v. injection of (+)-[^18^F]flubatine in mice, piglets, and humans, as presented in this work, to previous dosimetry data of an ex vivo biodistribution study (organ harvesting method) in 27 mice and PET/CT imaging of 6 piglets and 3 humans after i.v. injection of (−)-[^18^F]flubatine [16].

## Methods

### Synthesis of (+)-[^18^F]flubatine

A detailed description of the synthesis was published by Deuther-Conrad et al. [1], a fully automated radiosynthesis by Patt et al. [22] and a highly efficient ^18^F-radiolabeling based on the latest generation of trimethylammonium precursors by Smits et al. [15].

The automated radiosynthesis under full GMP conditions as described by Patt et al. [22] was used for the dosimetry investigations in mice, piglets, and humans. Enantiomeric pure (+)-[^18^F]flubatine was synthesized in 40 min with a radiochemical yield of 30 %, a radiochemical purity of >97 % and a specific activity of about 3000 GBq/μmol.

### Preclinical investigations

All animal experiments were approved by the respective Institutional Animal Care and Use Committee and by the regional administration Leipzig of the Free State of Saxony, Germany, and are in accordance with national regulations for animal research and laboratory care (§ 8 section 1 Animal protection act) as well as with the standards set forth in the eighth edition of Guide for the Care and Use of Laboratory Animals.

#### Mice

Three female CD1 mice (age = 12 weeks; weight = 30.1 ± 0.4 g) were housed in a temperature-controlled box with a 12:12 h light cycle at 26 °C. They underwent anesthesia (U-410, AgnTho's AB, Sweden) in a chamber using 4.0 % isoflurane in 60 % O_2_ and 40 % synthetic air (Gas blender 100, MCQ Instruments, Italy) until they were fully motionless demonstrated by the lack of pedal withdrawal reflex. Afterwards, they were positioned prone on the mouse imaging chamber and the anesthesia was reduced to 1.7 % isoflurane at 250 mL/min. The animals were imaged up to 4 h after i.v. injection of 9.4 ± 2.4 MBq (+)-[^18^F]flubatine into the lateral tail vein. The imaging chamber was maintained to keep the animals at 37 °C continuously to prevent hypothermia, and their breath frequency was controlled by a pneumatic pressure sensor at the chest.

#### Piglets

Three female piglets (age = 43 days; weight = 14 ± 1 kg) underwent anesthesia using 20 mg/kg ketamine, 2 mg/kg azaperone, 0.5 % isoflurane in 70 % N_2_O/30 % O_2_. Subsequently, they were artificially ventilated after surgical tracheotomy by a ventilator (Ventilator 710, Siemens-Elema, Sweden) followed by a 0.2 mg/kg/h pancuronium bromide i.v. injection. All incision sites were infiltrated with 1 % lidocaine. Volume substitution was supplied through a vein access (lactated Ringer’s solution, 5 mL/kg/h]). To monitor the arterial blood gas and acid-base parameters, blood samples were taken from the arteria femoralis with a polyurethane catheter to the aorta abdominalis and analyzed for relevant parameters (Radiometer ABL 50, Copenhagen, Brønshøj, Denmark). After finishing all surgical preparations, the concentration of anesthetic gas was reduced to 0.25 % isoflurane, 65 % N_2_O, and 35 % O_2_. The body temperature was monitored by an in-ear thermometer and maintained using a heating blanket throughout the imaging session. PET scans were sequentially conducted up to 5 h after i.v. (v. jugularis) injection of 183.5 ± 9.0 MBq (+)-[^18^F]flubatine.

### Clinical study

All experiments with (+)-[^18^F]flubatine were authorized by the responsible authorities in Germany, the Federal Institute for Drugs and Medical Devices (Bundesamt für Arzneimittel und Medizinprodukte, BfArM), the federal Office for Radiation Protection (Bundesamt für Strahlenschutz, BfS), the local ethics committee, and the institutional review board. All study participants gave their written consent to take part in the first-in-human study, particularly, that the data obtained can be analyzed scientifically including publication. The trial was registered in the EU clinical trials database, https://eudract.ema.europa.eu on 25/07/2012 with the trial registration number 2012-003473-26. The first subject was enrolled on 11/04/2014.

Three non-smoking healthy volunteers (two males, one female; age = 58 ± 6 years; weight = 81 ± 6 kg) gave their written informed consent. During the screening procedure, the individual medical and surgical history, including medication and allergies, was documented. Furthermore, a physical examination of the major body systems (plus height and weight), including vital signs, ECG (12-lead) and blood as well as urine samples was performed. None of the three volunteers fulfilled any exclusion criteria (i.e., significant abnormal physical examination, evidence of any significant illness from history, clinical, or para-clinical findings). The subjects were sequentially imaged in a PET/CT system (Biograph16, SIEMENS, Erlangen, Germany) after i.v. injection of 285.9 ± 12.6 MBq (+)-[^18^F]-flubatine.

### Instrumentation

#### Preclinical PET/MRI

The mouse studies were performed using a commercially available preclinical PET/MRI system (nanoScan®, MEDISO Budapest, Hungary). The three-dimensional lutetium-yttrium-orthosilicate (LYSO) 12 PS-PMT based PET-detector system with an axial field of view (FOV) of 94.7 mm has a resolution of 1.5–2.0 mm (transaxial) and 1.3–1.6 mm (axial) [23]. Each PET data set was corrected for random coincidences, dead time, scatter, and attenuation correction (AC), based on a whole-body MR scan segmented into soft tissue and air. The reconstruction parameters were as follows: 3D-ordered subset expectation maximization (OSEM), four iterations, six subsets, energy window 400–600 keV.

#### Clinical PET/CT

The piglet and human studies were performed on the clinical PET/CT system as mentioned above using a low-dose CT-AC and iterative PET reconstruction (OSEM, four iterations, eight subsets).

Both systems are subjected to periodically detector normalization and activity calibration. Furthermore, all peripheral devices to be used for the investigation (dose calibrator, gamma counter) are cross calibrated in terms of timing and radioactivity adjustment.

### PET scan protocol and data acquisition/evaluation

#### Mice

**Fig. 1:**
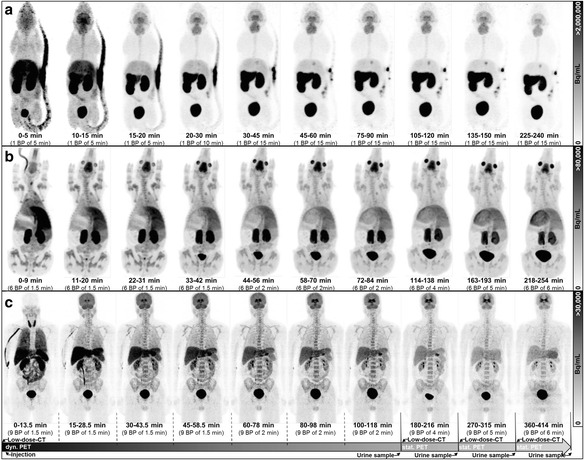
Dynamic PET image series (MIP) of mice (**a**), piglets (**b**), and humans (**c**). Each series shows the tracer accumulation in whole brain and particularly in the striatum followed by a washout through the hepatobiliary and the renal system. The animals did not void during the course of imaging. The healthy volunteers were asked to void as indicated in the timeline of the human investigational protocol

The animals were positioned prone in a special mouse imaging chamber (MultiCell, MEDISO Budapest, Hungary), with the head fixed to a mouth piece for the anesthetic gas supply. The PET data was collected in list-mode by a continuous whole-body (WB) scan during the entire investigation using the whole FOV at one bed position (BP). Subsequently, the list-mode-data was rebinned into sinograms of time frames (3 × 5 min, 1 × 10 min, 6 × 15 min) up to 240 min. p.i. (Fig. 1). Following the PET scan, a T1-weighted WB gradient echo sequence (TR = 20 ms; TE = 3.2 ms) was performed for anatomical orientation and segmentation of a μ-map (soft tissue and air) for AC.

#### Piglets

The piglets were positioned prone with their legs alongside the body on a custom-made plastic trough including a special head-holder. The PET acquisition was divided in a sequential (4 × 9 min, 3 × 12 min) and a static part (1 × 24 min, 1 × 30 min,1 × 36 min) (Fig. 1) each of which was preceded by a low-dose CT for AC.

#### Humans

The subjects were positioned supine with their arms down. The PET acquisition was similar to that for piglets but with an extended timeline for the sequential (4 × 13.5 min, 3 × 18 min) and static part (1 × 36 min, 1 × 45 min, 1 × 54 min) (Fig. 1). The subjects left the system during the examination to stretch out and for voiding the urinary bladder. The volume of all urine p.i. was measured and its activity concentration determined by sampling 3 × 0.5 ml in tubes to be measured with a gamma-counter (Cobra II 5003, PACKARD, 3-in NaI crystal). The activity concentration was decay corrected to the actual time of micturition.

### Image analysis

All images were analyzed with the Software package ROVER (ABX, Radeberg, Germany; v. 2.1.17) [24]. The organs that exhibited tracer uptake were identified and manually delineated using 3D volumes of interest (VOI). The MR and CT images were used for anatomical orientation and for image registration with the PET data. Source organs are the brain, gallbladder, large intestine, small intestine, stomach, heart, kidneys, liver, lungs, pancreas, red marrow (backbone, pelvis, sternum), spleen, thyroid, testes, and urinary bladder. The activity data of each VOI was assigned to the individual organ or tissue and subsequently transformed into percentage of injected dose (%ID_organ_) with Eq. 1 [16],

1 $$ \%{\mathrm{ID}}_{{\mathrm{organ}}_t}=\frac{A_{{\mathrm{organ}}_t}\cdot {c}_{{\mathrm{scan}}_t}}{A_{t(0)}}\left[\%\right] $$

where ${A}_{{\mathrm{organ}}_t}$ is the activity in the organ at the corresponding time *t*, ${c}_{{\mathrm{scan}}_t}$ is an image calibration factor representing differences between imaged and injected activity which is calculated from the injected wholebody activity decay corrected to *t* and divided by the imaged body activity (whole body mask delineated by threshold so that the ROI volume equals the body weight of the animal/volunteer assuming a tissue density of 1 g/cm3) of the respective image time frame, and *A*_*t*(0)_ is the injected activity decay corrected to *t*_0_.

### Dose calculation

Due to differences in weight, size, and metabolic rates between the smaller animal species and the human volunteers, it is necessary to map the preclinical (mouse and piglet) extracted biodistribution data to the human circumstances. Thus, the animal biokinetic data (time scale and %ID values) was adapted to the human circumstances using Eqs. 2 and 3 to fit the human weight, size, and metabolic rates [25].

The allometric metabolic rate scaling was found in physiological processes between species to be close to 1/4 for example when comparing blood flow per cycle of mice (15 s [26]) and rats (15 s [27]) to humans (50 s [28, 29]). Therefore, the following equation was chosen for time adaption to humans:

2 $$ {t}_{\mathrm{human}}={t}_{\mathrm{animal}}{\left\{\frac{m_{\mathrm{human}}}{m_{\mathrm{animal}}}\right\}}^{0.25} $$

where *t*_animal_ is the animal timescale, *t*_human_ is the human time scale, and $\frac{m_{\mathrm{human}}}{m_{\mathrm{animal}}}$ is the ratio between animal and human body weights.

Among other mass scaling methods, the %ID/g method [30] has been widely applied:

3 $$ {\left(\frac{\%\mathrm{ID}}{\mathrm{organ}}\right)}_{\mathrm{human}}={\left(\frac{\%\mathrm{ID}}{\mathit{\mathsf{g}}}\right)}_{\mathrm{animal}}{\left({m}_{\mathrm{organ}}\right)}_{\mathrm{human}}\cdot \frac{m_{\mathrm{animal}}}{m_{\mathrm{human}}} $$

where $\frac{\%\mathrm{ID}}{{\mathrm{organ}}_{\mathrm{human}}}$ is the fraction of the injected activity in the corresponding human organ, $\frac{\%\mathrm{ID}}{{\mathit{\mathsf{g}}}_{\mathrm{animal}}}$ is the fraction of injected activity per gram animal organ tissue, and ${m}_{{\mathrm{organ}}_{\mathrm{human}}}$ is the mass of the corresponding human organ (e.g., hermaphrodite model as implemented in OLINDA). The scaled biokinetic data served as input into the EXM module in the OLINDA software [31]. The time-activity curves were fitted by exponential curves (least mean squares fit), and the area under the curve was calculated giving the total number of disintegrations (NOD) occurring in the respective organ during the time of investigation.

**Fig. 2:**
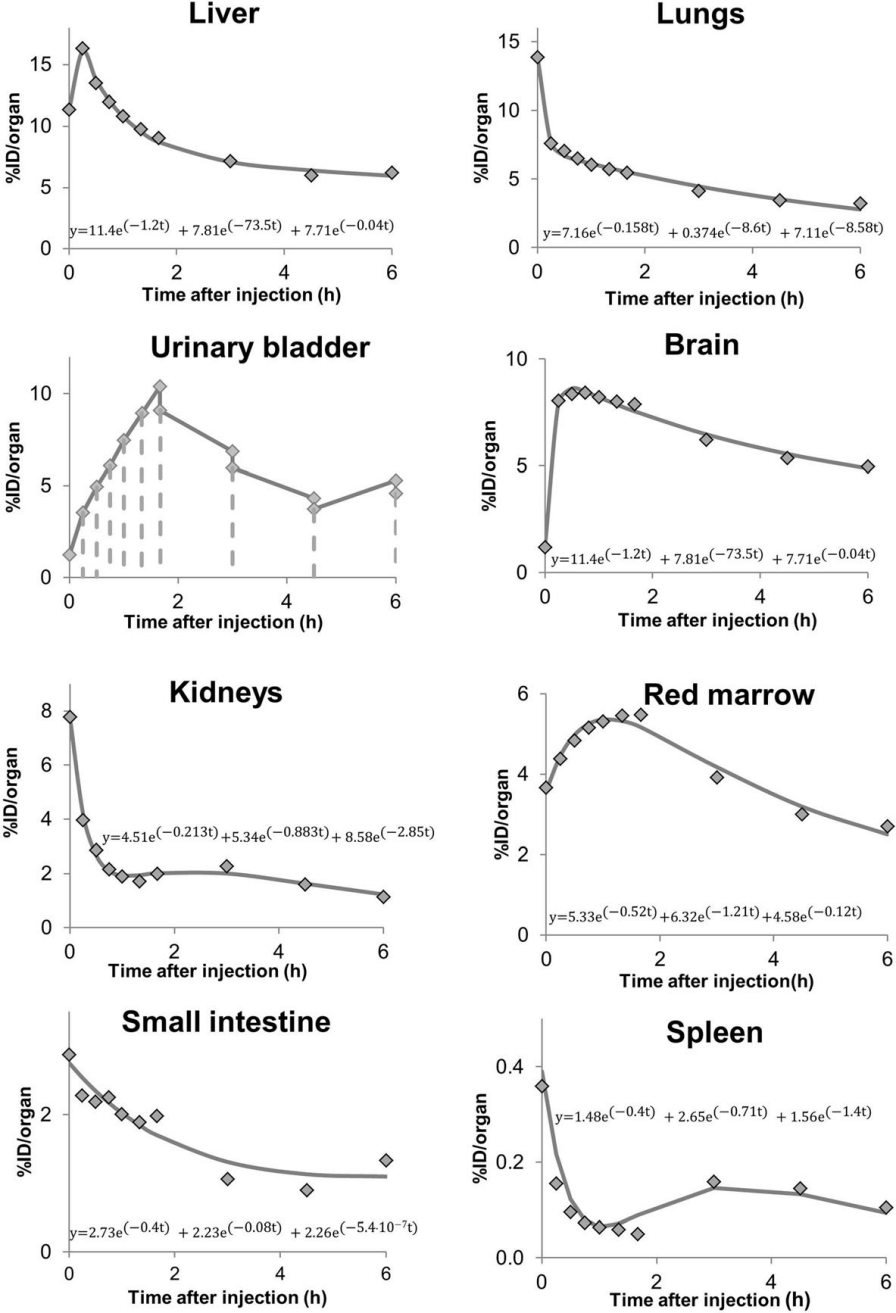
Exemplary time-activity curves of one human subjects and the respective exponential fit functions for different organs. For the urinary bladder, combined image and urine sample activity concentration data were used and the number of disintegrations is determined using the trapezoidal fit

Mice and piglets did not void urine during the investigation time, and thus, the curve fits of the urinary bladder were done using the activity data from PET imaging. The voided urine from human and therefore the decrease of activity needs to be included into the calculation in another way. As fitting as well as the voiding bladder model does not represent the time course of activity in the urinary bladder very well, the integral (=NOD) of the time-activity curve in the human urinary bladder was calculated by the trapezoidal equation [32] (Eq. 4). This gives a better approximation of the real course of activity in the human urinary bladder and, thus, the cumulated activity in it (Fig. 2).

4 $$ \%{\overset{\sim }{\mathrm{ID}}}_{\mathrm{UB}}=\frac{1}{2}{\displaystyle {\sum}_{i=1}^{n-1}\left(\%{\mathrm{ID}}_i+\%{\mathrm{ID}}_{i+1}\right)}\left({t}_{i+1}-{t}_i\right) $$

where *%*ID_*i,*_ is the fraction of injected activity at time *t*_*i*_ and $\%{\tilde{\mathrm{ID}}}_{\mathrm{UB}}$ is the cumulated activity of the urinary bladder, i.e., the NOD. However, the dose of the UB when using the International Commission on Radiological Protection (ICRP) dynamic bladder model is displayed in brackets for the human study in the “Results” and “Discussion” sections. The mean absorbed organ doses were estimated using the adult male phantom [33] based on the mapped to human animal biodistribution data or human data. To assess the overall risk in humans, the effective dose (ED) concept was used by multiplying the organ absorbed doses (OD) with the tissue weighting factors as published in the ICRP 103 [34]. However, as these weighting factors requires the ICRP 110 phantom [35] which is not available in OLINDA v.1.0, the ED results by using the tissue weighting factors published in ICRP 60 [36] can be found in the last table row (Tables 1, 2, and 3).

**Table 1:** ODs and ED for (+)-[^18^F]flubatine based on mice data

Target organ	OD	SD	ED contribution	SD
	(mSv/MBq)		(mSv/MBq)	
Adrenals	1.19E−02	2.02E−03	1.02E−04	1.74E−05
Brain	1.3E−02	5.77E−05	1.33E−04	5.77E−07
Breasts	7.24E−03	1.94E−03	8.68E−04	2.33E−04
Gallbladder wall	1.49E−02	6.43E−04	1.28E−04	5.53E−06
LLI wall	1.35E−02	1.15E−03	8.10E−04	6.92E−05
Small intestine	1.96E−02	2.81E−03	1.69E−04	2.42E−05
Stomach wall	1.47E−02	7.94E−04	1.76E−03	9.52E−05
ULI wall	2.05E−02	3.44E−03	1.23E−03	2.06E−04
Heart wall	9.75E−03	2.34E−03	8.38E−05	2.01E−05
Kidneys	4.75E−02	2.17E−02	4.08E−04	1.87E−04
Liver	2.05E−02	6.38E−03	8.21E−04	2.55E−04
Lungs	7.87E−03	1.81E−03	9.45E−04	2.17E−04
Muscle	9.02E−03	2.04E−03	7.76E−05	1.75E−05
Ovaries	1.22E−02	1.68E−03	9.76E−04	1.35E−04
Pancreas	1.41E−02	1.95E−03	1.21E−04	1.68E−05
Red marrow	9.95E−03	1.79E−03	1.19E−03	2.15E−04
Osteogenic cells	1.42E−02	3.42E−03	1.42E−04	3.42E−05
Skin	6.95E−03	1.73E−03	6.95E−05	1.73E−05
Spleen	1.41E−02	6.01E−03	1.21E−04	5.17E−05
Testes	8.84E−03	1.97E−03	–	–
Thymus	8.62E−03	2.27E−03	7.41E−05	1.95E−05
Thyroid	9.41E−03	1.41E−03	3.77E−04	5.65E−05
Urinary bladder wall	3.34E−02	1.68E−02	1.34E−03	6.72E−04
Uterus	1.28E−02	1.31E−03	1.10E−04	1.13E−05
Total body	9.87E−03	1.95E−03	–	–
ED (mSv/MBq)			1.21E−02	6.97E−04
ED (mSv/MBq) ICRP 60			1.29E−02	8.29E−04

ODs calculated for the adult male model (73.7 kg, implemented in OLINDA) based on mouse biodistribution and organ geometry data that were scaled to human circumstances

*OD* organ dose, *ED* effective dose (ICRP 103), *SD* standard deviation mean over three animals

**Table 2:** ODs and ED for (+)-[^18^F]flubatine based on piglet data

Target organ	OD	SD	ED contribution	SD
	(mSv/MBq)		(mSv/MBq)	
Adrenals	1.11E−02	7.64E−04	9.57E−05	6.57E−06
Brain	3.23E−02	3.24E−03	3.23E−04	3.24E−05
Breasts	6.21E−03	5.15E−04	7.45E−04	6.18E−05
Gallbladder wall	1.81E−02	1.18E−03	1.56E−04	1.02E−05
LLI wall	1.15E−02	4.04E−04	6.88E−04	2.42E−05
Small intestine	1.48E−02	1.00E−03	1.27E−04	8.61E−06
Stomach wall	1.10E−02	6.51E−04	1.32E−03	7.81E−05
ULI wall	1.52E−02	1.15E−03	9.12E−04	6.92E−05
Heart wall	1.42E−02	2.15E−03	1.22E−04	1.85E−05
Kidneys	4.51E−02	6.50E−03	3.88E−04	5.59E−05
Liver	2.69E−02	4.78E−03	1.07E−03	1.91E−04
Lungs	1.39E02	2.00E−04	1.67E−03	2.40E−05
Muscle	7.77E−03	4.03E−04	6.68E−05	3.46E−06
Ovaries	1.07E−02	2.08E−04	8.53E−04	1.67E−05
Pancreas	2.24E−02	1.78E−03	1.93E−04	1.53E−05
Red marrow	1.15E−02	1.07E−03	1.38E−03	1.28E−04
Osteogenic cells	1.35E−02	1.21E−03	1.35E−04	1.21E−05
Skin	5.87E−03	4.13E−04	5.87E−05	4.13E−06
Spleen	2.01E−02	9.07E−04	1.73E−04	7.80E−06
Testes	7.70E−03	6.51E−05	–	–
Thymus	1.82E−02	3.27E−03	1.56E−04	2.81E−05
Thyroid	1.77E−02	8.90E−03	7.07E−04	3.56E−04
Urinary bladder wall	7.17E−02	2.63E−02	2.87E−03	1.05E−03
Uterus	1.28E−02	1.01E−03	1.10E−04	8.73E−06
Total body	9.39E−03	7.64E−04	–	–
ED (mSv/MBq)			1.43E−02	2.60E−04
ED (mSv/MBq) ICRP 60			1.52E−02	5.0E−04

ODs calculated for the adult male model (73.7 kg, implemented in OLINDA) based on piglet biodistribution and organ geometry data that were scaled to human circumstances

*OD* organ dose, *ED* effective dose (ICRP 103), *SD* standard deviation mean over three animals

**Table 3:** ODs and ED for (+)-[^18^F]flubatine based on human data

Target organ	OD	SD	ED contribution	SD
	(mSv/MBq)		(mSv/MBq)	
Adrenals	1.30E−02	2.26E−03	1.12E−04	1.94E−05
Brain	2.86E−02	3.33E−03	2.86E−04	3.33E−05
Breasts	6.29E−03	3.86E−04	7.55E−04	4.63E−05
Gallbladder wall	2.34E−02	9.30E−03	2.01E−04	7.99E−05
LLI wall	1.67E−02	2.89E−03	1.00E−03	1.74E−04
Small intestine	2.94E−02	8.90E−03	2.53E−04	7.66E−05
Stomach wall	2.04E−02	3.89E−03	2.44E−03	4.67E−04
ULI wall	3.24E−02	1.02E−02	1.94E−03	6.14E−04
Heart wall	1.57E−02	1.65E−03	1.35E−04	1.42E−05
Kidneys	3.81E−02	4.52E−03	3.28E−04	3.89E−05
Liver	5.31E−02	2.98E−02	2.12E−03	1.19E−03
Lungs	2.70E−02	2.60E−03	3.24E−03	3.12E−04
Muscle	7.90E−03	1.26E−04	6.80E−05	1.08E−06
Ovaries	1.31E−02	1.22E−03	1.05E−03	9.73E−05
Pancreas	2.08E−02	4.11E−03	1.79E−04	3.53E−05
Red marrow	1.49E−02	8.50E−04	1.79E−03	1.02E−04
Osteogenic cells	1.42E−02	7.81E−04	1.42E−04	7.81E−06
Skin	5.52E−03	1.27E−04	5.52E−05	1.27E−06
Spleen	1.34E−02	4.19E−03	1.15E−04	3.60E−05
Testes	2.17E−02	1.21E−02	1.53E−03	1.33E−03
Thymus	7.48E−03	2.91E−04	6.43E−05	2.50E−06
Thyroid	2.29E−02	4.37E−03	9.17E−04	1.75E−04
Urinary bladder wall	1.02E−01	2.96E−02	4.09E−03	1.18E−03
Uterus	1.55E−02	1.71E−03	1.33E−04	1.47E−05
Total body	1.05E−02	8.42E−04	–	–
ED (mSv/MBq)			2.30E−02	1.91E−03
ED (mSv/MBq) ICRP 60			2.48E−02	1.18E−03

ODs calculated for the adult male model (73.7 kg, implemented in OLINDA)

*OD* organ dose, *ED* effective dose (ICRP 103), *SD* standard deviation mean over three human subjects

## Results

In this study, we have investigated the preclinical dosimetry of (+)-[^18^F]flubatine, a radiotracer for imaging of α_4_β_2_ nAChRs, by in vivo PET imaging of mice and piglets. The biokinetic data was extrapolated to the human scale and the ODs and ED estimated with OLINDA. In a subsequent first-in-human study in three healthy volunteers, the radiation safety was confirmed which supports the translation of this promising radioligand into further clinical phases.

After i.v. injection of (+)-[^18^F]flubatine, no adverse effects were observed in the investigated species based on vital signs monitoring. A 60 min p.i. WB PET image of a mouse, piglet, and an adult is presented in Additional file 1: Figure S1, showing high uptake in the brain, liver, stomach, and urinary bladder. An example of the manually delineated VOIs using the MR (Additional file 1: Figure S2) and CT information is shown in Additional file 1: Figure S3 (mice, piglet, human). The WB PET series in Fig. 1 for all three species show, among others, the differences of urine excretion as described above. Furthermore, the tracer accumulation in the liver, kidneys, and brain followed by a washout can be observed. The calculated ODs and ED based on the animal data and estimated for the adult male model are presented as mean values for 25 organs in Tables 1, 2, and 3 for mice, piglets, and humans, respectively. Detailed biokinetic data expressed as %ID can be found in Additional file 1: Table S1 to S3.

### Estimation of the radiation exposure in humans from preclinical imaging data

The high OD absorbed by the urinary bladder, kidneys, and liver indicate the major pathways of tracer elimination through the renal and the hepatobiliary system followed by a fast washout from the remaining organs (Fig. 1). The highest ODs were observed (values in μSv/MBq for mice and piglets) in the urinary bladder (33.4 ± 16.8, 71.7 ± 26.3), the kidneys (47.5 ± 21.7, 45.1 ± 6.5), and the liver (20.5 ± 6.4, 26.9 ± 4.8). The ED calculated from biokinetic animal data mapped to humans is 12.1 ± 0.7 μSv/MBq (mice) and 14.3 ± 0.3 μSv/MBq (piglets). Exemplary time-activity curves for (+)-[^18^F]flubatine are presented in Additional file 1: Figure S4 to S6 for mice, piglets, and humans, respectively, compared to previous results after application of (−)-[^18^F]flubatine. The time-activity curves (TACs) of both enantiomers from the piglet study are similar for almost all organs. Hence, no significant difference (students *t* test: piglets *p* = 0.77) was found for the assessment of the ED. However, methodological issues between the imaging and harvesting method cause differences among the two tracers investigated in the mice studies.

### First-in-human study, human internal dosimetry

There were no adverse effects reported in any of the three volunteers after i.v. injection of (+)-[^18^F]flubatine, and no significant changes in vital signs were monitored. Representative time-activity curves were plotted and their exponential fit functions calculated and exemplarily shown for the human dosimetry in Fig. 2 for eight organs. The highest ODs values were estimated in the urinary bladder (102.4 ± 29.6 or 28.8 ± 21.5 when applying the ICRP voiding bladder model with 4 h voiding interval), kidneys 38.1 ± 4.5, and liver 53.1 ± 29.8. The ED of (+)-[^18^F]flubatine after i.v. injection in human volunteers was estimated to be 23.0 ± 1.9 μSv/MBq.

## Discussion

In this study, we have shown the radiation safety of (+)-[^18^F]flubatine in preclinical studies prior to a first-in-human clinical trial. Furthermore, we demonstrated the feasibility of small animal PET/MR imaging to estimate the radiation dose in humans based on mice biokinetic image data scaled to human anatomy. However, the presented study confirmed that preclinical internal dosimetry underestimates the ED in humans by 38–47 % as it has already been shown for (−)-[^18^F]flubatine [16] and other radiotracers [37–40].

The main findings of the current investigation are (i) a reproducible ED result for (+)-[^18^F]flubatine compared to (−)-[^18^F]flubatine in humans based on extrapolated biokinetic data from mice and piglets, (ii) differences in the biodistribution in mice between the two enantiomers of [^18^F]flubatine due to methodological issues, (iii) shortcomings regarding radiation dose estimates for humans based on extrapolated small animal data, (iv) a better dose estimation in humans when using larger species to collect biokinetic data which is extrapolated to human scale, and (v) the confirmation of the radiation safety of (+)-[^18^F]flubatine for clinical studies.

### Preclinical PET/MRI and PET/CT for dose estimation in humans

Our previous study of (−)-[^18^F]flubatine showed that internal dosimetry based on biokinetic data acquired by sacrificing mice, organ harvesting, and measuring organs in a gamma-counter results in an ED of 12.5 μSv/MBq. In the current study, we determined the biodistribution of (+)-[^18^F]flubatine with the same species (similar parameters of breed, age, weight, sex) but using image-based activity quantification with a preclinical PET/MRI system. The mice organs could be clearly identified and manually delineated by using the structural MR information as shown in Additional file 1: Figure S2. Thereby, the ED was estimated to be 12.1 μSv/MBq following i.v. injection of (+)-[^18^F]flubatine. This is slightly lower but not significantly different (*p* = 0.44, *t* test) than assessed by the organ harvesting method. Bretin et al. [41] found a similar deviation of the ED as determined using preclinical image based dosimetry (ED: [^18^F]FDOPA: 16.8 μSv/MBq, ED: [^18^F]FTYR: 15.0 μSv/MBq) and organ harvesting (ED: [^18^F]FDOPA: 16.0 μSv/MBq, ED: [^18^F]FTYR: 14.3 μSv/MBq). However, large differences between the two enantiomers of [^18^F]flubatine can be observed in mice with regard to the biokinetic data (Additional file 1: Figure S4) and the ODs (Table 1), most probably due to the following substantial methodically difference in determining organ activities by the two preclinical methods. In contrast to the organ harvesting study, a limitation of the image-based method is that the animals are under isoflurane narcosis which is known to slow down the metabolism and influences the hemodynamics [42, 43]. As a result, the tracer uptake and subsequent wash-out differs between these methods which explain partially the varying ODs. Based on the currently available data, no mechanistic explanation can be provided. The preclinical dosimetry of both enantiomers based on biokinetic PET/CT data from piglets scaled to human anatomy shows similar organ %ID values (Additional file 1: Figure S5). Consecutively, the calculated ODs are identical for most organs. This recursively confirms that the differences in the results of the two small animal-based dosimetry approaches (organ harvesting or imaging method) are really due to methodological differences as described above.

In comparison to the extrapolated animal data, the radiation exposure as determined in the first-in-human trial shows a 1.6- to 1.9-fold higher ED. The reason for this underestimation when using small animal data for human dose estimation is related to the following methodological shortcomings of the extrapolation methods. First, using the adult male model implemented in OLINDA 1.0 assumes that the anatomical arrangement (i.e., the geometric relations of organs and tissues) of mice and piglets is identical to that in humans. Hence, the applied mass extrapolation in animals does not account for the different positions and sizes and, thus, different radiation interactions of the organs to each other as reflected by the S-values in the respective phantom. Using rodent specific models (available with OLINDA 2.0 [44]) to solve this problem remains to be assessed. Secondly, the widely applied time and mass extrapolations cancel out differences in body and organ weight but do not account for species-specific differences of the target expression in the respective organs. Hence, the extrapolated uptake values of the organs differ significantly among the species.

The dosimetry data of piglets show no significant difference of the ED (students *t* test: piglets *p* = 0.77) compared to the previous study with (−)-[^18^F]flubatine, while using the same method (clinical PET/CT system). Additionally, using piglets for human dose estimates improves the ED results slightly while an underestimation of 38 % remains, mainly due to the limitations based on narcosis, time and mass scaling, the dosimetry phantom and species-species differences as discussed before. Although it seems that larger species can improve the human dosimetry based on animal data, Zanotti-Fregonara et al. [45] showed both under- and overestimation of the ED in humans ranging from −11 to +72 %, by using biokinetic data obtained in monkeys (weight 9.9 ± 3.6 kg). This gives further evidence to take species-specific pharmacokinetics of the radiotracers and metabolites into account as they result in significant deviations in preclinical dosimetry.

Despite the obvious shortcomings introduced by the organ mass and time scaling in preclinical internal dosimetry, these results justify the use of small animal image data to roughly estimate the radiation exposure in humans. Small animal PET/MR imaging yields comparable internal dosimetry results as the state-of-the-art organ harvesting method. Obviously, the main benefit of an image-based dosimetry with mice is not only the reduction of the necessary number of laboratory animals (3 versus up to about 30 per study) but also the reduction of study duration as well as tracer production resources. Furthermore, comparability of the results to those of piglet and human studies is increased, as the determination of the biodistribution and, thus, the dose calculations are based on quantitative PET-imaging as well.

### First-in-human study and dosimetry

The radiation dose by (+)-[^18^F]-flubatine in human tissues has been estimated after injection of the radiotracer in one female and two male healthy volunteers. The TACs (presented in Fig. 2) obtained hereby confirmed the renal/hepatobiliary clearance as assumed considering the preclinical results. Within 2 h p.i., the radioligand was rapidly cleared from the kidneys, liver, lungs, and spleen. The conversion factor of the effective dose by application of the α_4_β_2_ receptor ligand (+)-[^18^F]flubatine is 23.0 μSv/MBq and well within the range of what is known of other ^18^F-labeled diagnostic radiotracers. Hence, the application as PET imaging agent in humans is safe. Additional file 1: Figure S6 revealed similar biokinetics for both enantiomers of [^18^F]-flubatine based on %ID values. Therefore, the effective dose estimates show no significant difference (*p* = 0.77, *t* test). However, (+)-[^18^F]flubatine showed a higher affinity to α_4_β_2_ nAChRs compared to (−)-[^18^F]flubatine and a negligible amount of metabolites in our first-in-human study (data not shown). This supports the suggestion of the preclinical data that (+)-[^18^F]flubatine might be more sensitive detecting α_4_β_2_ nAChR reductions and that (+)-[^18^F]flubatine allows a quantification of α_4_β_2_ nAChRs without metabolite correction. Thus, in combination with the dosimetry, the application of (+)-[^18^F]flubatine in humans is safe and the further investigation as a clinical tool for PET imaging of α_4_β_2_ nAChRs is being analyzed in other parts of the aforementioned clinical trial.

## Conclusions

There is an underestimation of up to 47 % when using animal data for the assessment of the radiation exposure in humans although it was scaled with respect to mass and time scale differences between the species. It is of further evidence that allometric scaling can only compensate for the faster metabolism of the animals due to their size but not for actual differences in tracer uptake, species-specific target expression, and clearance in these species compared to humans. The dose conversion factor and the overall radiation risk represented by the effective dose is well within the range of what is known and well tolerated with other ^18^F-labeled tracers such as 19.0 μSv/MBq for [^18^F]FDG [46]. The deviation of preclinical dosimetry results from clinical study results suggests a systematic underestimation of the effective dose in humans below 50 % when determined using animal models.

## Additional file

Additional file 1:
**Figures S1** to **S6** and **Tables S1** to **S3**. **Figure S1.** PET images (maximum intensity projection, MIP) 1 h p.i. of a (A) mouse (29.7 g), (B) piglet (14.0 kg), and (C) human (77 kg). The accumulation in the brain, liver, urinary bladder, red marrow, and the intestines can be clearly identified in all three species. Compared with humans, there is an additional tracer uptake in the salivary gland in mice and in the eyes in piglets. **Figure S2.** T1 weighted gradient echo sequence (TR = 20 ms; TE = 3.2 ms; MIP) of a mouse. The high soft tissue contrast in the MRI allows for clear organ delineation. The heart, stomach, large intestines, small intestines, liver, and the coronary vessels can be clearly identified. Furthermore, a map of linear attenuation coefficients was segmented (soft tissue and air) from this image for scatter and attenuation correction. **Figure S3.** Exemplary PET images (MIP) of a mouse (A), piglet (B), and a female human (C) with VOIs highlighted. **Figure S4.** Organ by organ comparison of the mice imaging ((+)-[^18^F]flubatine) vs. organ harvesting ((−)-[^18^F]flubatine) method (mean %ID). **Figure S5.** Organ by organ comparison of the piglet imaging method after application of (−)- and (+)-[^18^F]flubatine (mean %ID). **Figure S6.** Organ by organ comparison of the human imaging method after application of (−)- and (+)-[^18^F]flubatine (mean %ID). **Table S1.** Mouse biokinetic small animal PET/MR based data and extrapolation to human circumstances in %ID per organ (mean %ID). **Table S2.** Piglet biokinetic PET/CT-based data and extrapolation to human circumstances in %ID per organ (mean %ID). Table S3. Human biokinetic PET/CT based data (mean %ID). (PDF 878 kb)

## Abbreviations

AC: Attenuation correction
BP: Bed position
ECG: Electrocardiography
ED: Effective dose
FOV: Field of view
HPLC: High-performance liquid chromatography
ICRP: International Commission on Radiological Protection
ID: Injected dose
MRT: Magnet resonance tomography
nAChRs: Nicotinic acetylcholine receptors
NCFHEB: Norchloro-fluoro-homoepibatidine
NOD: Number of disintegrations
OD: Organ dose
OSEM: Ordered subset expectation maximization
PET: Positron emission tomography
PS-PMT: Position-sensitive photomultiplier tube
ROI: Region of interest
SPECT: Single-photon emission computed tomography
VOI: Volume of interest
WB: Whole-body

## References

[1] Deuther-Conrad W, Patt JT, Lockman PR, Allen DD, Patt M, Schildan A, Ganapathy V, Steinbach J, Sabri O, Brust P (2008). Norchloro-fluoro-homoepibatidine (NCFHEB)—a promising radioligand for neuroimaging nicotinic acetylcholine receptors with PET. Eur Neuropsychopharm.

[2] Lindstrom JON, Anand R, Peng X, Gerzanich V, Wang FAN, Li Y (1995). Neuronal nicotinic receptor subtypes. Ann NY Acad Sci.

[3] Quik M, Wonnacott S (2011). α6β2* and α4β2* nicotinic acetylcholine receptors as drug targets for parkinson's disease. Pharmacol Rev.

[4] Brašić, JR, James R, Cascella N, Kumar A, Zhou Y, Hilton J, Raymont V, Crabb A, Guevara MR, Horti AG, Wong DF. Positron emission tomography experience with 2‐[^18^F]fluoro‐3‐(2(s)‐azetidinylmethoxy) pyridine (2‐[^18^F]FA) in the living human brain of smokers with paranoid schizophrenia. Synapse. 2012;66(4):352–68. 10.1002/syn.21520 PMC3445266 22169936

[5] Wu J, Ishikawa M, Zhang J, Hashimoto K. Brain imaging of nicotinic receptors in Alzheimer's disease. Int J Alzheimers Dis. 2010;2010:548913. doi:10.4061/2010/548913. 10.4061/2010/548913 PMC3022172 21253523

[6] Sabri O, Steinbach J, Wilke S, Brust P, Hoepping A, Smits R, Wagenknecht G, Becker G, Graef S, Hesse S (2012). PET imaging of cerebral nicotinic acetylcholine receptors (nAChRs) in early Alzheimer′s disease assessed with the new radioligand (−)-[^18^F]norchloro-fluoro-homoepibatidine ([^18^F]Flubatine). J Cerebr Blood F Met.

[7] Sabri O (2015). First-in-human PET quantification study of cerebral α4β2* nicotinic acetylcholine receptors using the novel specific radioligand (−)-[^18^F] Flubatine. Neuroimage.

[8] Fischer S, Hiller A, Smits R, Hoepping A, Funke U, Wenzel B, Cumming P, Sabri O, Steinbach J, Brust P (2013). Radiosynthesis of racemic and enantiomerically pure (−)-[^18^F]flubatine—a promising PET radiotracer for neuroimaging of α4β2 nicotinic acetylcholine receptors. Appl Radiat Isotopes.

[9] Kendziorra K, Wolf H, Meyer P, Barthel H, Hesse S, Becker GA, Luthardt J, Schildan A, Patt M, Sorger D, Sabri O (2011). Decreased cerebral α4β2* nicotinic acetylcholine receptor availability in patients with mild cognitive impairment and Alzheimer’s disease assessed with positron emission tomography. Eur J Nucl Med Mol I.

[10] Gallezot JD, Bottlaender M, Greoire MC, Roumenov D, Deverre JR, Coulon C, Ottaviani M, Dolle F, Syrota A, Valette H (2005). In vivo imaging of human cerebral nicotinic acetylcholine receptors with 2-[^18^F]-Fluoro-A-85380 and PET. J Nucl Med.

[11] Kant R, Constantinescu CC, Parekh P, Pandey SK, Pan ML, Easwaramoorthy B, Mukherjee J (2011). Evaluation of [^18^F]nifene binding to α4β2 nicotinic receptors in the rat brain using microPET imaging. Eur J Nucl Med Mol I.

[12] Deuther-Conrad W, Patt JT, Feuerbach D, Wegner F, Brust P, Steinbach J (2004). Norchloro-fluoro-homoepibatidine: specificity to neuronal nicotinic acetylcholine receptor subtypes in vitro. Farmaco.

[13] Brust P, Patt JT, Deuther‐Conrad W, Becker G, Patt M, Schildan A, Sabri O (2008). In vivo measurement of nicotinic acetylcholine receptors with [^18^F] norchloro‐fluoro‐homoepibatidine. Synapse.

[14] Hockley BG, Stewart MN, Sherman P, Quesada C, Kilbourn MR, Albin RL, Scott PJ (2013). (−)-[^18^F]flubatine: evaluation in rhesus monkeys and a report of the first fully automated radiosynthesis validated for clinical use. J Labelled Comp Radiopharm.

[15] Smits R, Fischer S, Hiller A, Deuther-Conrad W, Wenzel B, Patt M, Cumming P, Steinbach J, Sabri O, Brust P, Hoepping A (2014). Synthesis and biological evaluation of both enantiomers of [^18^F]flubatine, promising radiotracers with fast kinetics for the imaging of α4β2-nicotinic acetylcholine receptors. Bioorgan Med Chem.

[16] Sattler B, Kranz M, Starke A, Wilke S, Donat CK, Deuther-Conrad W, Patt M, Schildan A, Patt J, Smits R, Hoepping A, Schoenknecht P, Steinbach J, Brust P, Sabri O (2014). Internal dose assessment of (−)-[^18^F]flubatine, comparing animal model datasets of mice and piglets with first-in-human results. J Nucl Med.

[17] Patt M, Becker G, Grossmann U, Habermann B, Schildan A, Wilke S, Deuther-Conrad W, Graef S, Fischer S, Smits R, Hoepping A, Wagenknecht G, Steinbach J, Gertz HJ, Hesse S, Schoenknecht P, Brust P, Sabri O (2014). Evaluation of metabolism, plasma protein binding and other biological parameters after administration of (−)-[^18^F] Flubatine in humans. Nucl Med Biol.

[18] McParland BJ (2010). Nuclear medicine radiation dosimetry: advanced theoretical principles.

[19] McParland BJ (2010). Nuclear medicine radiation dosimetry: advanced theoretical principles.

[20] Constantinescu CC, Sevrioukov E, Garcia A, Pan ML, Mukherjee J (2013). Evaluation of [^18^F]mefway biodistribution and dosimetry based on whole-body PET imaging of mice. Mol Imaging Biol.

[21] Constantinescu CC, Garcia A, Mirbolooki MR, Pan ML, Mukherjee J (2013). Evaluation of [^18^F]nifene biodistribution and dosimetry based on whole-body PET imaging of mice. Nucl Med Biol.

[22] Patt M, Schildan A, Habermann B, Fischer S, Hiller A, Deuther-Conrad W, Wilke S, Smits R, Hoepping A, Wagenknecht G, Steinbach J, Brust P, Sabri O (2013). Fully automated radiosynthesis of both enantiomers of [^18^F]flubatine under GMP conditions for human application. Appl Radiat Isot.

[23] Nagy K, Tóth M, Major P, Patay G, Egri G, Häggkvist J, Varrone A, Farde L, Halldin C, Gulyás B (2013). Performance evaluation of the small-animal nanoScan PET/MRI system. J Nucl Med.

[24] Hofheinz F, Pötzsch C, Oehme L, Beuthien-Baumann B, Steinbach J, Kotzerke J, van den Hoff J (2012). Automatic volume delineation in oncological PET. Evaluation of a dedicated software tool and comparison with manual delineation in clinical data sets. Nuklearmed.

[25] Stabin MG (2008). Fundamentals of nuclear medicine dosimetry.

[26] Welsher K, Sherlock SP, Dai H (2011). Deep-tissue anatomical imaging of mice using carbon nanotube fluorophores in the second near-infrared window. P Natl Acad Sci USA.

[27] Lin JH (1998). Applications and limitations of interspecies scaling and in vitro extrapolation in pharmacokinetics. Drug Metab Dispos.

[28] Stabin MG (2008). Fundamentals of nuclear medicine dosimetry.

[29] McParland BJ (2010). Nuclear medicine radiation dosimetry: advanced theoretical principles.

[30] Kirschner AS, Ice RD, Beierwaltes WH (1975). Radiation dosimetry of ^131^I-19-iodocholesterol: the pitfalls of using tissue concentration data—reply. J Nucl Med.

[31] Stabin MG (2005). OLINDA/EXM: the second-generation personal computer software for internal dose assessment in nuclear medicine. J Nucl Med.

[32] Stabin MG (2008). Fundamentals of nuclear medicine dosimetry.

[33] Cristy M. Specific absorbed fractions of energy at various ages from internal photon sources. VII. Adult Male. Oak Ridge National Laboratory. 1987:Document Number ORNL/TM-8381/V7.

[34] ICRP (2007). The 2007 recommendations of the International Commission of Radiological Protection: ICRP publication 103s. Ann ICRP.

[35] ICRP. Adult Reference Computational Phantoms: ICRP Publication 110—Annals of the ICRP. Maryland Heights, MO: Elsevier; 2009; 39 (2):1–165. 10.1016/j.icrp.2009.09.001 19897132

[36] International Commission on Radiological Protection. ICRP Publication 60: 1990 Recommendations of the International Commission on Radiological Protection. Ann. ICRP 21 (1-3); 1991. 2053748

[37] Doss M, Kolb HC, Zhang JJ, Bélanger MJ, Stubbs JB, Stabin MG, Hostetler E, Alpaugh R, von Mehren M, Walsh J, Haka M, Mocharla V, Yu J (2012). Biodistribution and radiation dosimetry of the integrin marker ^18^F-RGD-K5 determined from whole-body PET/CT in monkeys and humans. J Nucl Med.

[38] Maddahi J, Czernin J, Lazewatsky J, Huang SC, Dahlbom M, Schelbert H, Devine M (2011). Phase I, first-in-human study of BMS747158, a novel ^18^F-labeled tracer for myocardial perfusion PET: dosimetry, biodistribution, safety, and imaging characteristics after a single injection at rest. J Nucl Med.

[39] Lazewatsky J, Azure M, Guaraldi M, Kagan M, MacDonald J, Yu M, Robinson S (2008). Dosimetry of BMS747158, a novel ^18^F-labeled tracer for myocardial perfusion imaging, in nonhuman primates at rest. J Nucl Med.

[40] Takano A, Gulyás B, Varrone A, Karlsson P, Sjoholm N, Larsson S, Hoffmann A (2011). Biodistribution and radiation dosimetry of the 18 kDa translocator protein (TSPO) radioligand [^18^F]FEDAA1106: a human whole-body PET study. Eur J Nucl Med Mol Imaging.

[41] Bretin F, Mauxion T, Warnock G, Bahri MA, Libert L, Lemaire C, Plenevaux A (2014). Hybrid microPET imaging for dosimetric applications in mice: improvement of activity quantification in dynamic microPET imaging for accelerated dosimetry applied to 6-[^18^F]fluoro-L-DOPA and 2-[^18^F]lluoro-L-tyrosine. Mol Imaging Biol.

[42] Alkire MTM, Haier RJP, Shah NKM, Anderson CTM (1997). Positron emission tomography study of regional cerebral metabolism in humans during isoflurane anesthesia. Anesthesiology.

[43] Toyama H, Ichise M, Liow JS, Vines DC, Seneca NM, Modell KJ, Innis RB (2004). Evaluation of anesthesia effects on [^18^F]FDG uptake in mouse brain and heart using small animal PET. Nucl Med Biol.

[44] Stabin M, Farmer A (2012). OLINDA/EXM 2.0: the new generation dosimetry modeling code. J Nucl Med.

[45] Zanotti-Fregonara P, Innis RB (2012). Suggested pathway to assess radiation safety of ^11^C-labeled pet tracers for first-in-human studies. Eur J Nucl Med Mol Imag.

[46] ICRP (2008). Radiation dose to patients from radiopharmaceuticals: (Addendum 3 to ICRP Publication 53) ICRP publication 106. Ann ICRP.

