# Supplementary material for: Radiation dosimetry of the α4β2 nicotinic receptor ligand (+)-[18F]flubatine, comparing preclinical PET/MRI and PET/CT to first-in-human PET/CT results
Source: EJNMMI Phys. 2016 Oct 21;3:25. doi: 10.1186/s40658-016-0160-5 (PMC5074934; doi:10.1186/s40658-016-0160-5)
Supplement: Additional file 1: — Figures S1 to S6 and Tables S1 to S3. Figure S1. PET images (maximum intensity projection, MIP) 1 h p.i. of a (A) mouse (29.7 g), (B) piglet (14.0 kg), and (C) human (77 kg). The accumulation in the brain, liver, urinary bladder, red marrow, and the intestines can be clearly identified in all three species. Compared with humans, there is an additional tracer uptake in the salivary gland in mice and in the eyes in piglets. Figure S2. T1 weighted gradient echo sequence (TR = 20 ms; TE = 3.2 ms; MIP) of a mouse. The high soft tissue contrast in the MRI allows for clear organ delineation. The heart, stomach, large intestines, small intestines, liver, and the coronary vessels can be clearly identified. Furthermore, a map of linear attenuation coefficients was segmented (soft tissue and air) from this image for scatter and attenuation correction. Figure S3. Exemplary PET images (MIP) of a mouse (A), piglet (B), and a female human (C) with VOIs highlighted. Figure S4. Organ by organ comparison of the mice imaging ((+)-[18F]flubatine) vs. organ harvesting ((−)-[18F]flubatine) method (mean %ID). Figure S5. Organ by organ comparison of the piglet imaging method after application of (−)- and (+)-[18F]flubatine (mean %ID). Figure S6. Organ by organ comparison of the human imaging method after application of (−)- and (+)-[18F]flubatine (mean %ID). Table S1. Mouse biokinetic small animal PET/MR based data and extrapolation to human circumstances in %ID per organ (mean %ID). Table S2. Piglet biokinetic PET/CT-based data and extrapolation to human circumstances in %ID per organ (mean %ID). Table S3. Human biokinetic PET/CT based data (mean %ID). (PDF 878 kb) [file 40658_2016_160_MOESM1_ESM.pdf]

SUPPLEMENTAL MATERIAL FOR MANUSCRIPT:

"Radiation Dosimetry of the $\alpha_4\beta_2$ Nicotinic Receptor Ligand (+)-[^{18}F]Flubatine, Comparing Preclinical PET/MRI and PET/CT to First-in-Human PET/CT Results"

Mathias Kranz, Bernhard Sattler, Solveig Tiepolt, Stephan Wilke Winnie Deuther-Conrad, Cornelius K. Donat, Steffen Fischer, Marianne Patt, Andreas Schildan, Jörg Patt, René Smits, Alexander Hoepping, Jörg Steinbach, Osama Sabri, Peter Brust

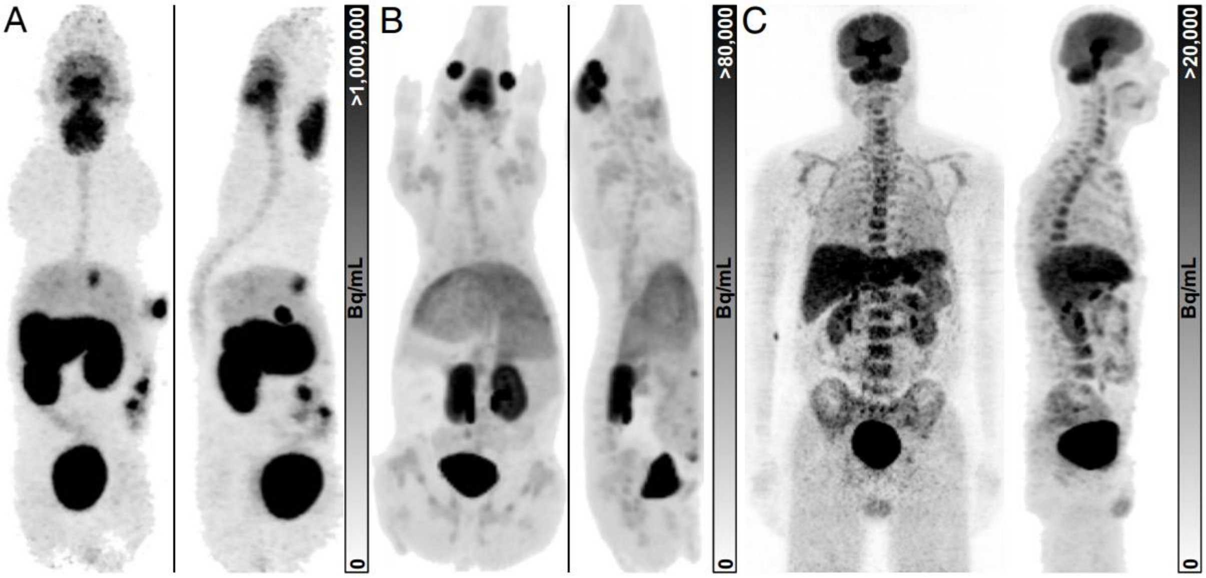

Figure S1. PET images (maximum intensity projection, MIP) 1h p.i. of a (A) mouse (29.7 g), (B) piglet (14.0 kg) and (C) human (77 kg). The accumulation in the brain, liver, urinary bladder, red marrow and the intestines can be clearly identified in all three species. Compared with humans there is an additional tracer uptake in the salivary gland in mice and in the eyes in piglets

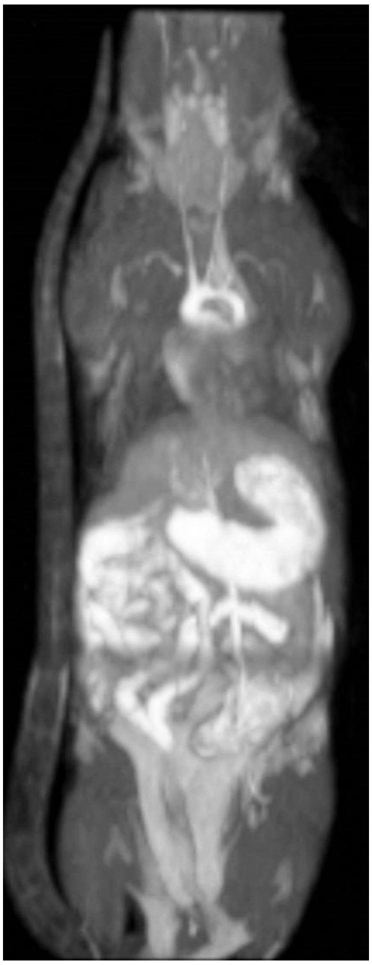

Figure S2. T_1 weighted gradient echo sequence (TR= 20 ms; TE= 3.2 ms; MIP) of a mouse. The high soft tissue contrast in the MRI allows for clear organ delineation. The heart, stomach, large intestines, small intestines, liver and the coronary vessels can be clearly identified. Furthermore, a map of linear attenuation coefficients was segmented (soft tissue and air) from this image for scatter- and attenuation correction

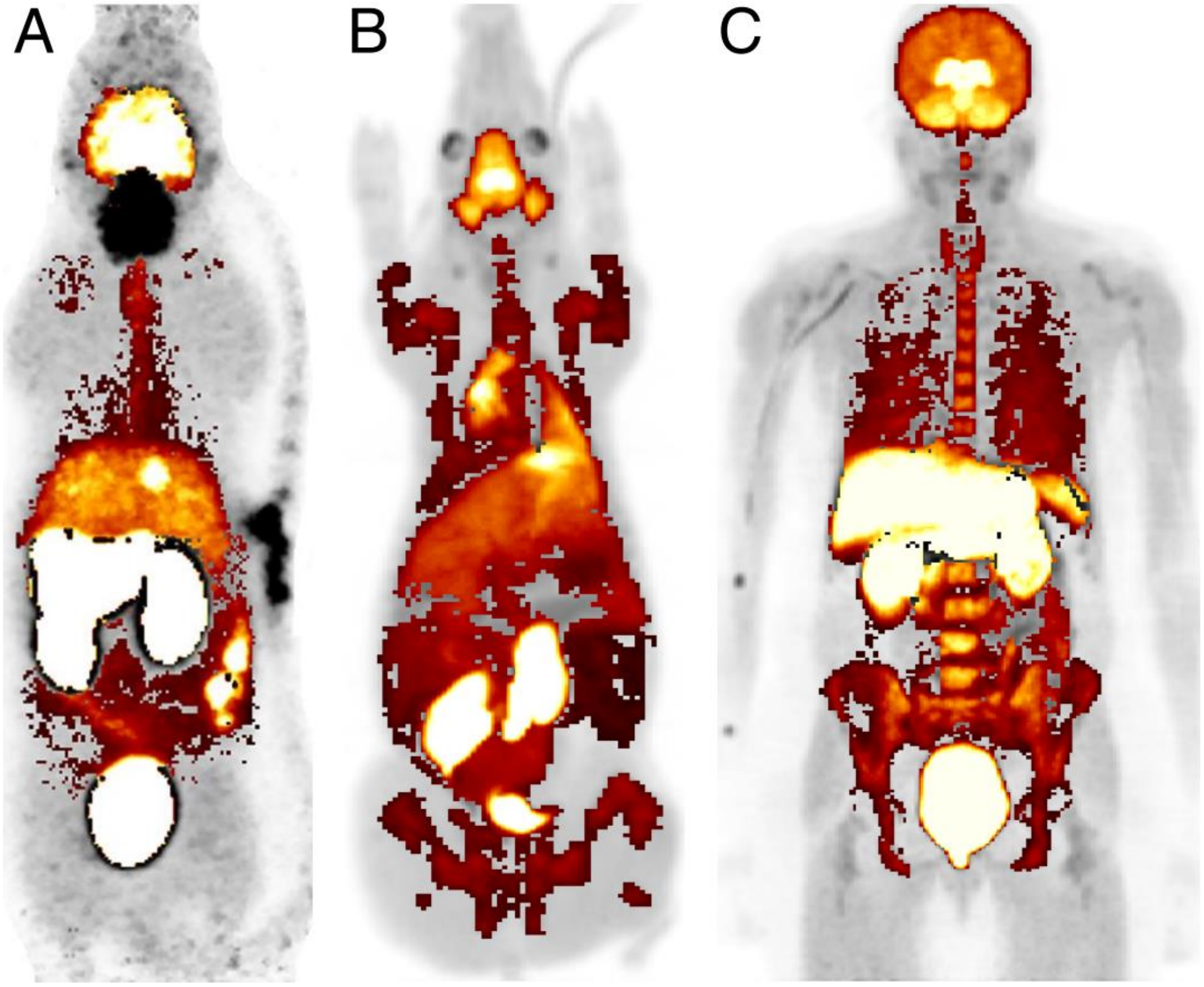

Figure S3. Exemplary PET images (MIP) of a mouse (A), piglet (B) and a female human (C) with VOIs highlighted

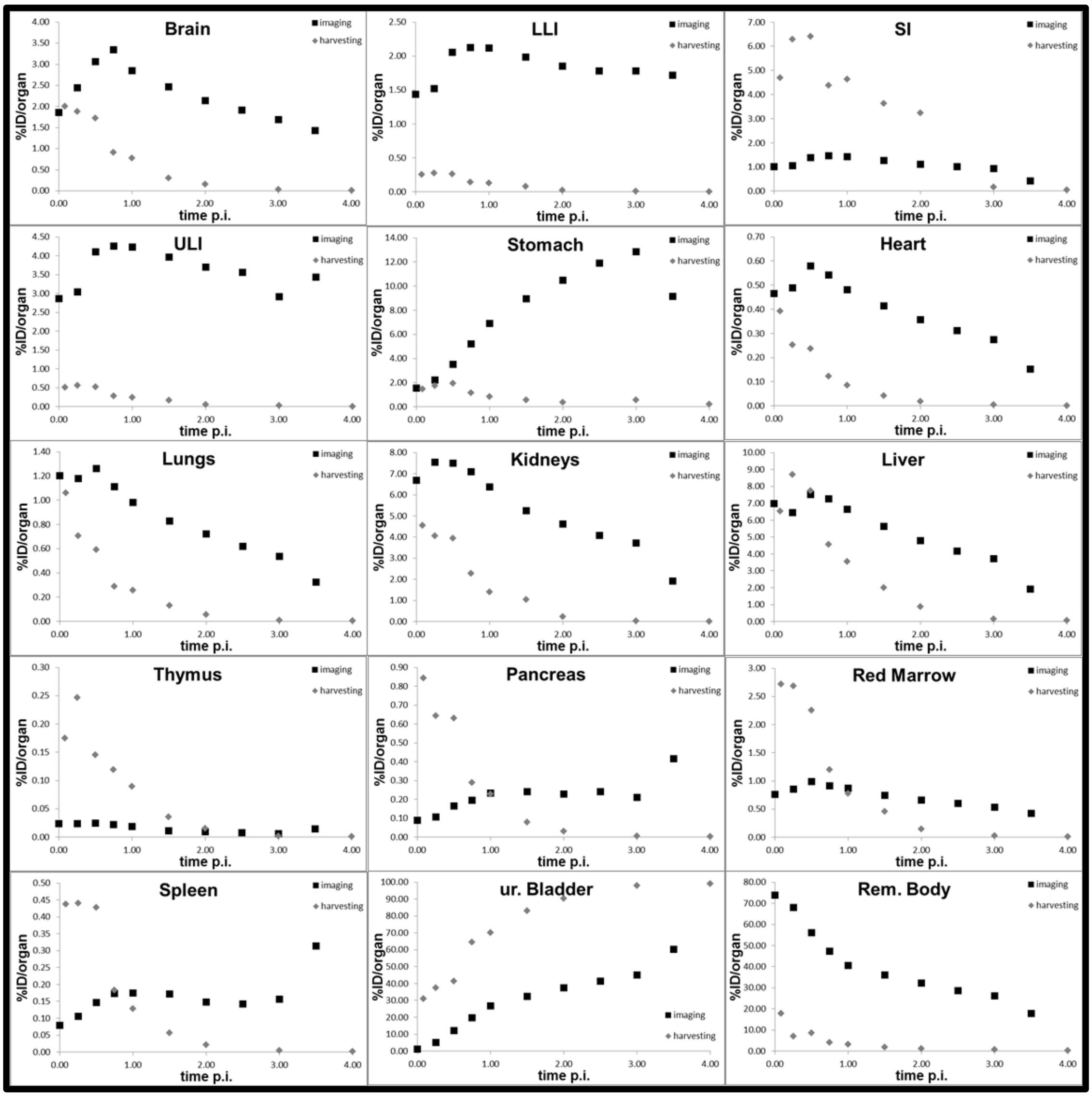

Figure S4. Organ by organ comparison of the mice imaging ((+)-[^{18}F]flubatine) vs. organ harvesting ((-)-[^{18}F]flubatine) method (mean %ID).

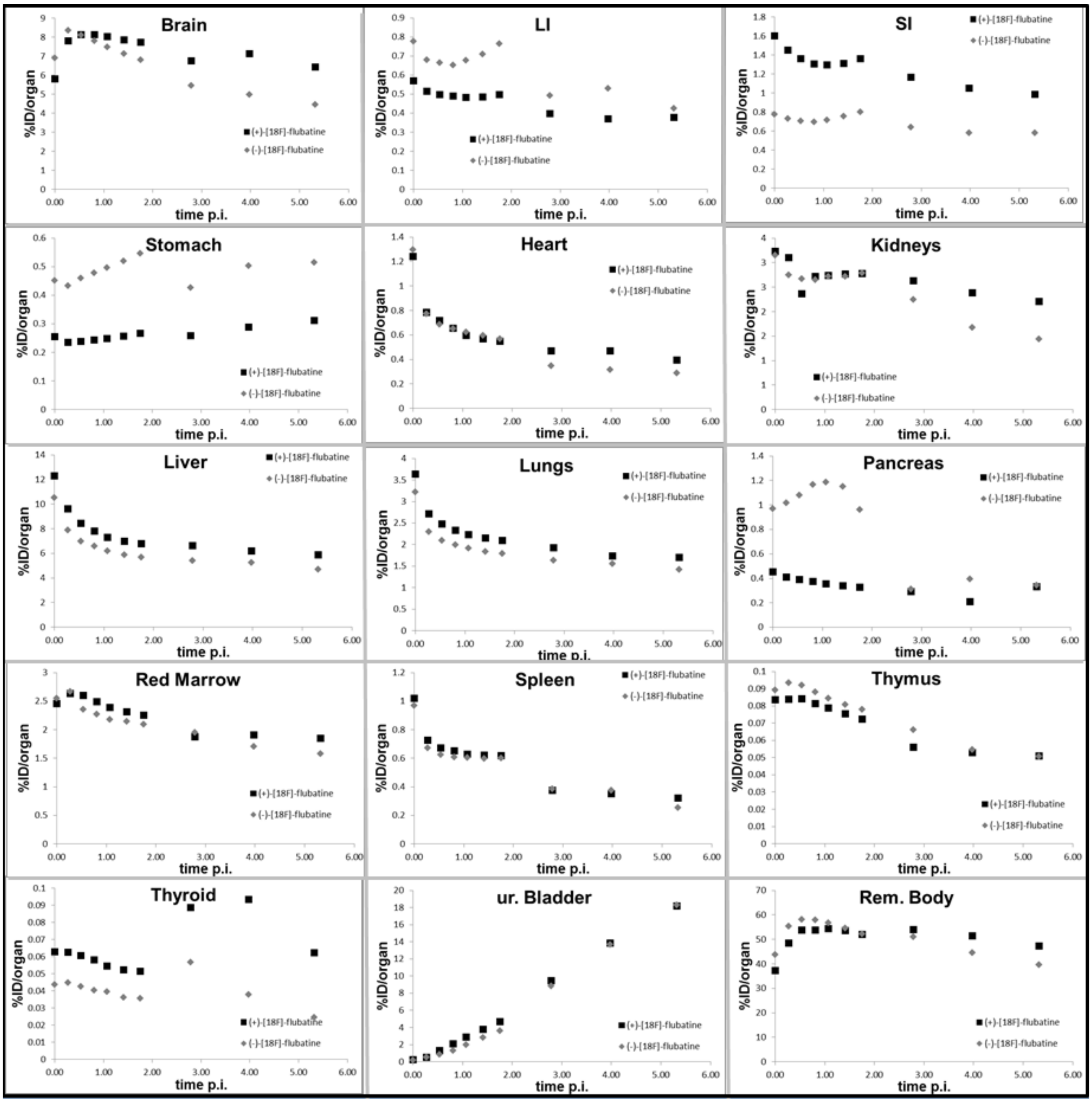

Figure S5.Organ by organ comparison of the piglet imaging method after application of (-)- and (+)-[^{18}F]flubatine (mean %ID).

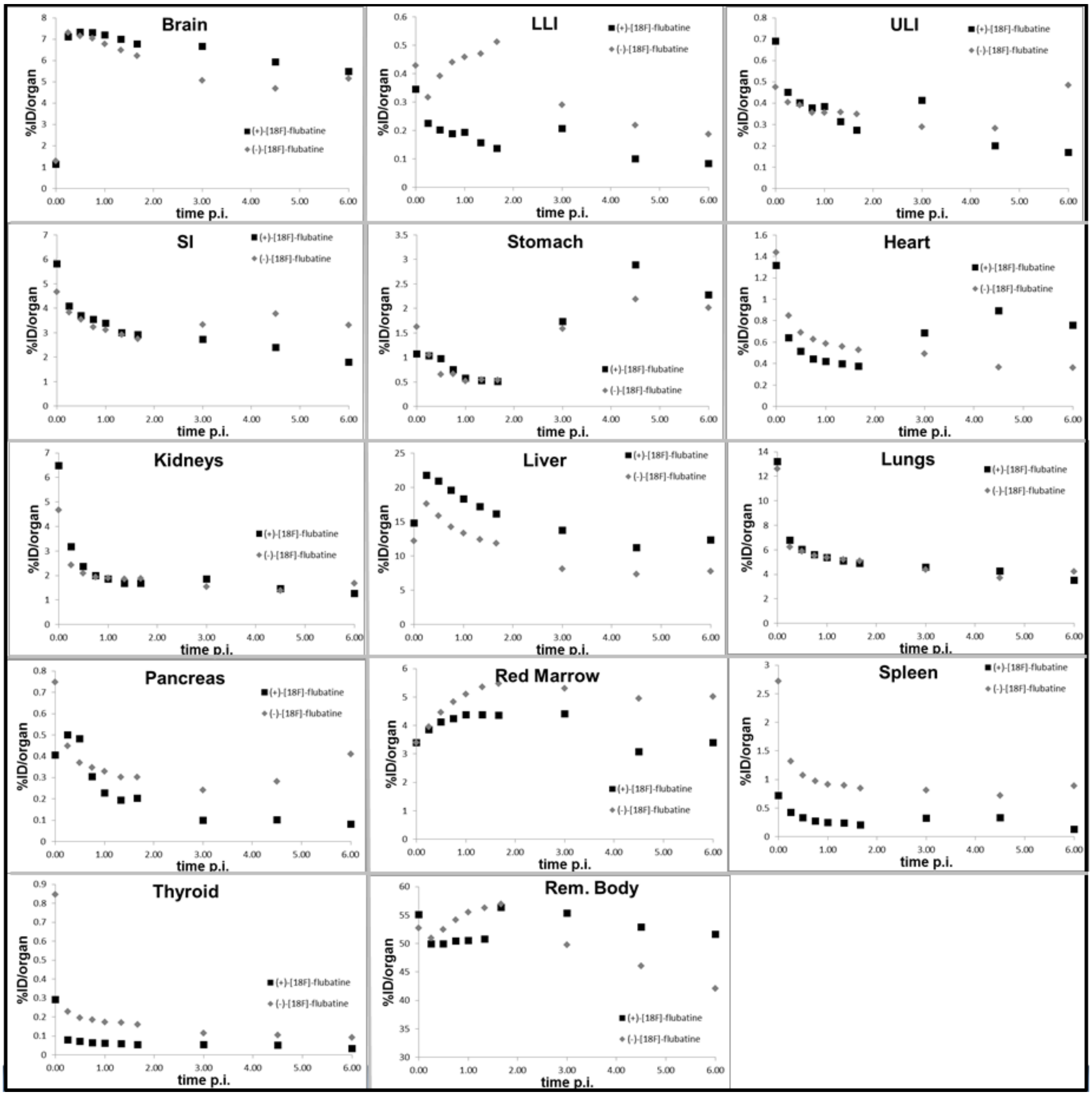

Figure S6.Organ by organ comparison of the human imaging method after application of (-)- and (+)-[^{18}F]flubatine (mean %ID).

Modell		Brain		LLI		SI		Stomach		ULI	
		animal	human	animal	human	animal	human	animal	human	animal	human
$\text{Time}_{\text{animal}}$[h]	$\text{Time}_{\text{human}}$ [h]	%ID/organ	%ID/organ	%ID/organ	%ID/organ	%ID/organ	%ID/organ	%ID/organ	%ID/organ	%ID/organ	%ID/organ
0.00	0.00	1.86	1.58	1.43	0.66	1.01	0.39	1.53	0.16	2.87	0.98
0.25	1.75	2.45	2.18	1.52	0.70	1.04	0.39	2.20	0.23	3.04	1.04
0.50	3.51	3.06	2.87	2.05	0.83	1.39	0.58	3.51	0.39	4.11	1.23
0.75	5.26	3.34	3.07	2.13	0.83	1.46	0.65	5.21	0.60	4.25	1.23
1.00	7.01	2.85	2.71	2.11	0.79	1.41	0.63	6.91	0.84	4.23	1.17
1.50	10.52	2.47	2.35	1.98	0.76	1.25	0.56	8.94	1.12	3.97	1.13
2.00	14.02	2.14	2.07	1.85	0.69	1.10	0.50	10.48	1.36	3.69	1.03
2.50	17.53	1.91	1.87	1.78	0.65	1.00	0.47	11.88	1.58	3.56	0.96
3.00	21.03	1.69	1.67	1.78	0.64	0.93	0.44	12.85	1.75	2.91	0.87
3.50	24.54	1.42	0.93	1.72	1.29	0.42	0.05	9.13	1.17	3.43	1.93

Heart		Kidneys		Liver		Lungs		Pancreas		Red Marrow	
animal	human	animal	human	animal	human	animal	human	animal	human	animal	human
%ID/organ	%ID/organ	%ID/organ	%ID/organ	%ID/organ	%ID/organ	%ID/organ	%ID/organ	%ID/organ	%ID/organ	%ID/organ	%ID/organ
0.47	0.16	6.69	2.33	6.99	4.58	1.20	0.66	0.09	0.08	0.76	0.51
0.49	0.17	7.55	2.67	6.44	4.45	1.18	0.65	0.11	0.08	0.86	0.62
0.58	0.21	7.50	2.80	7.52	5.39	1.26	0.68	0.16	0.12	0.99	0.79
0.54	0.19	7.08	2.68	7.25	5.25	1.11	0.60	0.19	0.14	0.91	0.73
0.48	0.17	6.37	2.43	6.65	4.79	0.98	0.53	0.23	0.16	0.87	0.71
0.41	0.15	5.25	2.00	5.63	4.00	0.83	0.44	0.24	0.15	0.75	0.61
0.36	0.13	4.62	1.76	4.79	3.39	0.72	0.38	0.23	0.14	0.66	0.55
0.31	0.11	4.07	1.55	4.17	2.91	0.62	0.33	0.24	0.15	0.60	0.50
0.27	0.10	3.72	1.55	3.72	2.56	0.54	0.28	0.21	0.12	0.54	0.45
0.15	0.05	1.91	0.72	1.91	1.78	0.32	0.21	0.42	0.17	0.42	0.17

Spleen		Thymus		Thyroid		ur. Bladder		Rem. of. Body	
animal	human	animal	human	animal	human	animal	human	animal	human
%ID/organ	%ID/organ	%ID/organ	%ID/organ	%ID/organ	%ID/organ	%ID/organ	%ID/organ	%ID/organ	%ID/organ
0.08	0.18	0.02	0.01	0.03	0.01	1.05	0.17	73.89	86.18
0.11	0.24	0.02	0.01	0.04	0.02	5.14	0.81	67.88	79.14
0.15	0.32	0.02	0.01	0.07	0.03	12.24	1.84	55.98	65.36
0.17	0.38	0.02	0.01	0.08	0.03	19.67	2.85	47.24	55.17
0.17	0.38	0.02	0.01	0.09	0.03	26.80	3.79	40.48	47.28
0.17	0.37	0.01	0.00	0.09	0.03	32.43	4.59	36.11	42.07
0.15	0.32	0.01	0.00	0.07	0.02	37.43	5.24	32.11	37.37
0.14	0.30	0.01	0.00	0.07	0.02	41.37	5.74	28.59	33.27
0.16	0.32	0.01	0.00	0.06	0.02	44.92	6.19	26.04	30.29
0.31	0.60	0.01	0.00	0.02	0.01	60.21	9.88	17.71	21.38

Table S1. Mouse biokinetic small animal PET/MR based data and extrapolation to human circumstances in %ID per organ (mean %ID).

Modell		Brain		SI		Stomach		ULI (2/3 of LI)		Heart	
		animal	human	animal	human	animal	human	animal	human	animal	human
$Time_{animal}$[h]	$Time_{human}$ [h]	%ID/organ	%ID/organ	%ID/organ	%ID/organ	%ID/organ	%ID/organ	%ID/organ	%ID/organ	%ID/organ	%ID/organ
9.00	0.0	1.2	5.8	9.6	1.6	5.4	0.3	7.2	0.6	0.9	1.2
9.00	0.3	1.6	7.8	8.8	1.4	5.0	0.2	6.4	0.5	0.5	0.8
9.00	0.6	1.7	8.1	8.3	1.4	5.0	0.2	6.2	0.5	0.5	0.7
9.00	0.8	1.7	8.1	8.0	1.3	5.1	0.2	6.1	0.5	0.5	0.7
12.00	1.1	1.7	8.0	8.0	1.3	5.2	0.2	6.1	0.5	0.4	0.6
12.00	1.5	1.7	7.9	8.2	1.3	5.4	0.3	6.1	0.5	0.4	0.6
12.00	1.8	1.6	7.7	8.6	1.4	5.6	0.3	6.3	0.5	0.4	0.5
24.00	2.9	1.4	6.7	6.9	1.2	5.4	0.3	5.1	0.4	0.3	0.5
30.00	4.1	1.5	7.1	6.1	1.1	6.1	0.3	4.6	0.4	0.3	0.5
36.00	5.5	1.3	6.4	5.8	1.0	6.5	0.3	4.8	0.4	0.3	0.4

Kidneys		Liver		Lungs		Pancreas		Red Marrow		Spleen	
animal	human	animal	human	animal	human	animal	human	animal	human	animal	human
%ID/organ	%ID/organ	%ID/organ	%ID/organ	%ID/organ	%ID/organ	%ID/organ	%ID/organ	%ID/organ	%ID/organ	%ID/organ	%ID/organ
9.0	3.2	15.8	12.3	11.7	3.6	0.5	0.5	8.3	2.5	1.2	1.0
8.6	3.1	12.4	9.6	8.8	2.7	0.4	0.4	8.8	2.6	0.8	0.7
6.6	2.4	10.9	8.4	8.0	2.5	0.4	0.4	8.7	2.6	0.8	0.7
7.6	2.7	10.1	7.8	7.5	2.3	0.4	0.4	8.4	2.5	0.8	0.7
7.7	2.7	9.4	7.3	7.2	2.2	0.4	0.4	8.0	2.4	0.7	0.6
7.8	2.8	9.0	7.0	6.9	2.2	0.3	0.3	7.8	2.3	0.7	0.6
7.9	2.8	8.7	6.8	6.8	2.1	0.3	0.3	7.6	2.3	0.7	0.6
7.4	2.6	8.5	6.6	6.2	1.9	0.3	0.3	6.2	1.9	0.4	0.4
6.6	2.4	8.0	6.2	5.6	1.7	0.2	0.2	6.4	1.9	0.4	0.4
6.1	2.2	7.6	5.9	5.5	1.7	0.3	0.3	6.1	1.9	0.4	0.3

Thymus		Thyroid		ur. Bladder		Rem. of. Body	
animal	human	animal	human	animal	human	animal	human
%ID/organ	%ID/organ	%ID/organ	%ID/organ	%ID/organ	%ID/organ	%ID/organ	%ID/organ
0.6	0.1	0.2	0.1	0.3	0.2	27.7	37.2
0.6	0.1	0.2	0.1	0.7	0.5	35.9	48.4
0.6	0.1	0.2	0.1	1.7	1.3	40.0	53.9
0.6	0.1	0.2	0.1	2.7	2.1	40.0	53.9
0.5	0.1	0.2	0.1	3.6	2.9	40.4	54.4
0.5	0.1	0.2	0.1	4.7	3.8	39.9	53.6
0.5	0.1	0.2	0.1	5.8	4.7	38.7	52.0
0.4	0.1	0.2	0.1	10.7	9.4	40.1	54.0
0.4	0.1	0.2	0.1	15.2	13.9	38.2	51.5
0.3	0.1	0.1	0.1	19.3	18.2	35.2	47.2

Table S2. Piglet biokinetic PET/CT based data and extrapolation to human circumstances in %ID per organ (mean %ID).

Time [h]	Brain %ID	Gallbladder %ID	LLI %ID	SI %ID	Stomach %ID	ULI %ID	Myocardium %ID	Kidneys %ID	Liver %ID	Lung %ID	Pancreas %ID
0.00	1.1	0.1	0.3	5.5	1.0	0.7	1.3	6.3	14.3	12.8	0.4
0.25	7.1	0.1	0.2	4.1	1.0	0.5	0.6	3.2	21.7	6.8	0.5
0.50	7.3	0.1	0.2	3.7	1.0	0.4	0.5	2.4	20.9	6.0	0.5
0.75	7.3	0.1	0.2	3.5	0.8	0.4	0.4	2.0	19.6	5.6	0.3
1.00	7.2	0.1	0.2	3.4	0.6	0.4	0.4	1.9	18.3	5.4	0.2
1.32	7.0	0.1	0.2	3.0	0.5	0.3	0.4	1.7	17.2	5.1	0.2
1.63	6.8	0.1	0.1	2.9	0.5	0.3	0.4	1.7	16.1	4.9	0.2
2.92	6.0	0.2	0.2	2.4	1.7	0.4	0.6	1.7	12.7	4.1	0.1
4.42	5.4	0.1	0.1	2.2	2.7	0.2	0.8	1.3	10.6	3.9	0.1
5.92	5.1	0.2	0.1	1.7	2.0	0.2	0.7	1.2	11.8	3.2	0.1

red marrow %ID	Spleen %ID	Thyroid %ID	ur. Bladder %ID	Rem. of Body %ID
3.3	0.7	0.3	2.2	52.0
3.8	0.4	0.1	5.4	47.3
4.1	0.3	0.1	8.2	47.4
4.2	0.3	0.1	10.3	48.2
4.4	0.2	0.1	12.4	47.9
4.4	0.2	0.1	14.5	48.2
4.4	0.2	0.1	16.2	48.3
3.9	0.3	0.0	8.7	40.6
2.8	0.3	0.0	7.9	37.8
3.0	0.1	0.0	9.9	36.9

Table S3. Human biokinetic PET/CT based data (mean %ID).
